# Intellectual property rights over ‘integrated’ medical devices: the potential health impacts and bioethical implications of rightsholders’ control

**DOI:** 10.1093/medlaw/fwaf001

**Published:** 2025-03-06

**Authors:** Aisling M McMahon, Opeyemi I Kolawole

**Affiliations:** School of Law and Criminology, Maynooth University, Maynooth, Ireland; School of Law and Criminology, Maynooth University, Maynooth, Ireland

**Keywords:** access to health, bioethics, intellectual property rights, medical devices, patents, copyright

## Abstract

Despite extensive literature examining intellectual property rights (IPRs) and access to health, there has been limited examination of how IPRs can potentially impact the development, access to, delivery of, and use of *medical devices*. This article fills this gap, focusing on patent and copyright protections applicable to elements of medical devices that are attachable to or implanted into the human body, such as prostheses or pacemakers. Although the human body itself is not patentable in Europe (Article 5, Biotechnology Directive), elements of medical devices created outside the body are patentable. Moreover, certain aspects of such medical devices can be subject to copyright, and other types of IPRs. This article provides an overview of the types of IPRs that can apply over attachable and implantable medical devices. Following this, and focusing specifically on copyright and patent rights, it argues that such IPRs, alongside incentivizing technological development in certain contexts, also give rightsholders significant control over key aspects of how individuals use and access IP-protected elements of such devices, with the potential for health-related impacts and bioethical implications. Accordingly, the article argues that greater understanding and scrutiny are needed within the health law and bioethics communities around the potential impacts of IPRs over medical devices.

## I. INTRODUCTION

The human body is not patentable in Europe.^1^ However, various elements of emerging ‘technologies’, including medical device technologies are patentable, even where such devices are intended to become part of the human body.^2^ Similarly, other intellectual property rights (IPRs) do not apply to the human body itself, but may apply over certain aspects of medical devices attached to or which operate within the body. This article provides an overview of the main types of IPRs that can apply specifically over intangible aspects of ‘medical devices’ that are *integrated* with the human body, that is devices that are attachable to the human body, such as limb prostheses, or devices which are implantable within the human body, such as pacemakers.^3^ It focuses on the role of patents and copyright law in this context, because such IPRs are often the most relevant IPRs in terms of their potential to be used in a way that can impact the development, access, and use of such devices. Building on the work of Quigley and Aydihongbe on the challenges for law posed by the integration of persons and goods in the medical device context including challenges for intellectual property rights, and upon prior work by one of the authors on the bioethical issues posed by how patents can apply over technologies related to how we treat, use and modify the human body more generally,^4^ this article argues that how IPRs can be used over aspects of integrated medical device technologies needs far deeper consideration within the health law and bioethics communities because it can give rise to a range of health impacts with the potential to lead to bioethical implications, including implications for device-users’ autonomy and dignity interests.^5^ Rightsholders’ use of IPRs over such devices can also, in some cases, limit healthcare practitioners’ and healthcare systems’ clinical autonomy by impacting the types of medical devices that are accessible.

In making such arguments, this article makes two key contributions. First, although there is extensive literature on the implications of patents for access to medicines, there is a dearth of literature on IPRs and access to *medical devices*.^6^ Moreover, there is limited literature on this issue written specifically for a health law and bioethics audience, which examines in detail how IPRs can apply to medical devices, and the potential ways that the grant and/or the existence and use of IPRs over various aspects of medical devices may give rise to potential health implications. This article fills this gap by probing how IP protections could apply to elements of medical devices and highlighting how certain uses of such rights can pose access to health implications.^7^ Secondly, and relatedly, this article demonstrates the potential for such IPRs, focusing on patent and copyright law, to be used in a manner that can impact the development, access, delivery, and use of medical devices, as well as highlighting some of the related bioethical implications that can arise. This analysis is intended to serve as a starting point for others within the health law and bioethics communities to probe further the role of IPRs in the medical device context. It calls for a deeper and more nuanced examination of the potential role of IPRs as a tool to incentivize the development of medical devices in certain contexts, and how this can (or should), be considered alongside the potential bioethical implications that can arise.

The article is structured as follows: Section II outlines the broad range of ‘technologies’ that are considered as ‘medical devices’. It defines what is meant by ‘integrated’ medical devices for the purposes of this article, and provides a justification for the focus on IPRs over integrated devices. Section III provides an overview of the main types of IPRs that can apply over integrated medical devices. This overview is provided to enable broader consideration of the role of IPRs over medical devices within the health law and bioethics communities. As this overview is directed specifically at this audience, it assumes limited knowledge of IPRs. Thus, it is framed in general terms and explains key IPR principles where relevant to medical devices to encourage greater engagement with such issues by these communities in the future. This overview provides the necessary foundations for the analysis that follows. Section IV demonstrates how rightsholders’ use of such IPRs—focusing specifically on patent and copyright protections—can potentially impact the access, delivery, development, and use of integrated medical devices, with knock-on bioethical and health implications. Section V concludes by arguing that, given these implications, the use of IPRs over integrated medical devices (and their component parts) requires greater examination within health law and bioethics contexts.

## II. IPRs OVER CONNECTABLE AND NON-CONNECTABLE MEDICAL DEVICES

The relationship between IPRs and access to health is contested, and we recognize that patents and other types of IPRs can have both an incentivizing function and can also be used in ways that impact access to health. For instance, there is considerable literature examining IPRs and access to medicines. On the one hand, some authors argue that IPRs are necessary to incentivize the development of new medicines.^8^ For example, Scherer highlights evidence indicating that the IP system has an important role in the research and development investment decisions of health technology manufacturers.^9^ This is because the IP system, particularly the exclusivity of the patent system,^10^ provides a pathway for manufacturers to generate an income stream from a technology, which offers a means to secure a return on their investment.^11^ In making this point, this article is not suggesting IPRs are the only or most efficient incentivization tool for the development of new health-technologies. Instead, IPRs typically provide incentives for certain types of innovation in certain contexts, such as where they are driven by market demand and the ability of markets to pay.^12^ This health innovation model, however, can – and does – lead to inequities and exclusions; for example, the IPR based incentives model can contribute to limited innovation for medicines for conditions primarily affecting low- and middle-income countries.^13^ However, a critical analysis of these issues is beyond the scope of this article; instead, here, it is taken that IPRs can be a key factor to incentivize certain types of innovation.

On the other hand, the exclusive powers conferred on rightsholders by IPRs can be used in a manner that creates barriers for access to medicines, as the exclusivity of IPRs enables rightsholders to charge high prices for medicines and other health-technologies, which may as a result be inaccessible for those who need access.^14^ How patents and other IPRs are used can also impact the available supplies of medicines and vaccines, and who can obtain such medicines and vaccines first.

Yet, despite considerable discussion of IPRs and access to medicines and vaccines, there has been limited scrutiny over how IPRs can affect access, delivery, and use of medical devices.^15^ Such issues received greater attention during the COVID-19 pandemic,^16^ particularly when reports emerged that attempts to address shortages of ventilator equipment for COVID-19 patients using 3D-printed parts could be threatened by IP infringement claims.^17^ These claims were later refuted.^18^ However, as one of us has argued elsewhere, it is legally plausible that IPRs could be used in such a manner, regardless of the health impacts, which demonstrates the need for scrutiny of this area.^19^ This article offers an examination of the multi-faceted role and impact of IPRs over medical devices focusing on the potential impacts of IPRs over how *integrated* medical devices are developed, accessed and used.

Before delving into the types of IPRs that arise over integrated medical devices, this section outlines what the term ‘medical device’ ordinarily encompasses, how the term ‘integrated’ medical device is defined for the purposes of this article, and the rationale for taking this focus.

### A. ‘Medical device’: Definitional scope within existing European frameworks

There is no standardized definition for ‘medical device’, and where specific definitions have been adopted, including within legislation and literature on ‘medical devices’, a broad definition is often used, thereby accommodating an expansive range of devices.^20^ For example, the European Union’s Regulation on Medical Devices (Regulation (EU) 2017/745) describes a ‘medical device’ as:

…*any* instrument, apparatus, appliance, software, implant, reagent, material or other article *intended by the manufacturer* to be used, alone or in combination, for *human beings* for one or more of the following specific medical purposes:

- diagnosis, prevention, monitoring, prediction, prognosis, treatment or alleviation of disease,
- diagnosis, monitoring, treatment, alleviation of, or compensation for, an injury or disability,
- investigation, replacement or modification of the anatomy or of a physiological or pathological process or state,
- providing information by means of in vitro examination of specimens derived from the human body, including organ, blood and tissue donations,

and which does not achieve its principal intended action by pharmacological, immunological or metabolic means, in or on the human body, but which may be assisted in its function by such means. [Emphasis added]^21^

Thus, within this European regulatory context, ‘medical device’ is an umbrella term that captures a range of instruments, machines, and tools, ranging from syringes to pacemakers.^22^ The World Health Organization (WHO) adopts a similar broad definition.^23^ Aronson and others describe a ‘medical device’ as a ‘contrivance designed and manufactured for use in health and not solely medicinal or nutritional’.^24^ Racchi and others state that ‘medical devices’ ‘are very wide-ranging products, such as apparatus/instruments, software and materials (ie substances)’.^25^ The expansive nature of ‘medical devices’ as a classification is demonstrated by the fact that software programmes,^26^ or 3D bio-printed tissues,^27^ can be classified as medical devices in certain circumstances.

A range of factors are relevant for medical device classification. For instance, medical devices are often distinguishable from pharmaceuticals by how they achieve their therapeutic functions,^28^ as under relevant EU laws, medical devices do not achieve their objectives ‘by pharmacological, immunological or metabolic means’.^29^ The WHO adopts similar distinguishing features.^30^ Moreover, the *intention of the manufacturer* of the device is important in determining classification as a medical device.^31^ A detailed examination of how medical devices are classified is beyond the scope of this article. It suffices here to recognize that the term ‘medical device’ captures a wide range of different types of devices that interact with the human body in a myriad of different ways.

### B. ‘Integrated’ medical devices and the human body

This article focuses specifically on ‘medical devices’ that are *integrated* with the human body. This notion of ‘integration’ draws on Quigley and Ayihongbe’s conception of the ‘integrated’ nature of persons and goods in the context of how certain medical devices can become integrated with, or become part of, the human body.^32^ More specifically, for the purposes of this article, we are using the term ‘integrated’ to mean *medical devices that are attachable to the human body or implantable in the human body, where such devices are intended to alter or augment the appearance or functions of the human body*. Thus, the implications of IPRs over non-attachable and non-implantable medical devices, such as stethoscopes, are beyond the scope of this article.

This article focuses specifically on IPRs that can apply over *integrated* medical devices for two reasons: First, integrated medical devices are intended to be attached to or implanted within the human body and will often seek to alter, functionally or aesthetically, an element/aspect of the human body. In some instances, such as in the case of a pacemaker the operation of such devices can have significant health implications. Moreover, how IPRs operate and can be used over such devices, as will be discussed further below, can, in certain contexts, impact how such devices are developed, accessed, used, and modified. Thus, it will be argued that IPRs and how such rights are used can have health-related implications, with knock-on potential bioethical implications including for individual’s autonomy (and access to such devices) and dignity interests.^33^ 

Secondly, and relatedly, although the human body is not patentable in Europe, integrated medical devices are patentable and can also be subject to other forms of IPRs. Integrated medical devices are created in a technical manner outside the human body, yet when they become integrated with the body (by being implanted or attached to the body), they could be seen as becoming a part of the human body.^34^ This gives pause for thought on the extent to which the patentability of—and use of patents over—such devices is coherent with Article 5 of the Biotechnology Directive, which stipulates that the human body itself is unpatentable.^35^ Further scrutiny is thus needed over the effects and broader health and bioethical implications of the grant or use of patents (and use of other IPRs) over such ‘technologies’, which this article provides.

Furthermore, the article uses two sub-categories of integrated medical devices, namely: (i) non-connectable integrated medical devices and (ii) connectable integrated medical devices. Under category (i), the term ‘non-connectable’ integrated device refers to devices that operate with no connection to an external control system outside the body. These devices are intended to be attached to or implanted in the human body, for example, to maintain the healthy functioning of the human body, or to fulfil primarily cosmetic purposes.^36^ Examples include but are not limited to heart stents, artificial joints, certain types of prosthetics, etc. These devices will often be controlled manually by the physical actions of the device-user or have a merely aesthetic function. Category (ii) refers to *connectable* integrated medical devices, that is devices that are connected to or controlled by an external device or source, which collects, stores, and/or transmits data to a third party that controls how such data are used and, potentially, can affect or impact how the device functions. Examples of connectable integrated medical devices include artificial pancreas systems, pacemakers, etc. Such connectable devices rely on an external source to operate, which is often controlled by the rightsholder or manufacturer, such as via a mobile phone app—whose software code underpinning its operation may be protected by IPRs held by the rightsholder. The link between the device and manufacturer or rightsholder is often ongoing for connectable devices, even after such devices are implanted or attached to the human body.

These further classifications—connectable and non-connectable integrated medical devices—are used in this article. This is because IPRs may apply differently to elements of connectable versus non-connectable integrated medical devices due to the differing nature of these technologies and the varying levels of ongoing control that rightsholders have over the day-to-day functioning of a device in these two contexts.

## III. IPRs AND INTEGRATED MEDICAL DEVICES: AN OVERVIEW

This section provides a general overview of the main types of IPRs that can arise over elements of integrated medical devices in Europe and how these rights, in general terms, can be used by rightsholders over connectable and non-connectable medical devices.^37^ This provides the foundations for analysing the potential health and bioethical implications posed by how IPRs can be used over such devices, examined in Section IV. Importantly, multiple types of IPRs often apply over different aspects of a medical device, which may be held by different rightsholders, and as in other contexts, rightsholders may seek to strategically layer different types of IPRs to strengthen the exclusivity they have over an invention, a point discussed further below. Indeed, given how the IPR system is designed and operates, it can be expected that many rightsholders (often for-profit companies) would seek to maximize the breadth and length of their proprietary protection and to strategically use IPRs in this way. In making such arguments, we are not seeking to question whether IPRs should be granted over medical devices (or their component parts) *per se*. Instead, our focus is on illuminating the breadth of discretion rightsholders have once patents are granted (or other types of IPRs apply) over medical devices, which enables such IP rightsholders to exert considerable control over various aspects of how the IP-protected technologies are developed, used, and accessed.

Turning to the main types of IPRs applicable over integrated medical devices, the following key categories can be identified:

### A. Patents and integrated medical devices

A patent allows rightsholders to exclude others from using a patented invention, within the jurisdiction that the patent is granted in, for a minimum of 20 years.^38^ Prior to delving into how patents apply over elements of medical devices, a note on jurisdictional scope is needed. There is no global patent system. However, the TRIPS Agreement sets out minimum standards around patentability, which applies in all World Trade Organization Member States. Alongside this, national and regional laws apply depending on the jurisdiction. This article focuses on the European context. For patents, ‘European’ is used to mean the laws applicable under the European Patent Convention 1963, as amended (EPC), which apply to 39 States, including all European Union (EU) states and several non-EU States. The EU adopted the Biotechnology Directive 98/44/EC, which applies to the patentability of biotechnologies in the EU, and this Directive was adopted as supplementary interpretation for the EPC. Thus, for patents, this section refers primarily to the TRIPS Agreement, EPC and, where relevant, the Biotechnology Directive. The institutional complexities of the European patent system are beyond the scope of this article.^39^

Article 27(2) of the TRIPS Agreement (and Article 52(1) EPC) states that patents must be made available for inventions in all fields of technology, provided that the invention is new, involves an inventive step and is capable of industrial application.^40^

Thus, medical device technologies or related technologies are patentable under TRIPS. Moreover, a patent can be granted for both a final product (or element(s) of that product) and the process(es) of making/using that product. Furthermore, a rightsholder can hold patents over the product (or elements of that product) and over a related process simultaneously. Under European patent law^41^ and the international TRIPS framework,^42^ certain inventions are not patentable. However, there is no exclusion from patentability against medical devices *per se*; instead, medical devices are treated the same as other patentable technologies, regardless of the purpose of the underlying patented ‘technology’ and how the device is intended to interact with or operate within (or in relation to) the human body.^43^ Indeed, once a patent is granted, patentable inventions are treated as fungible within the patent system, and ‘[n]o consideration is made of the effects of such patents and the associated licenses consequent upon the nature of the “invention” in question’.^44^

Moreover, whilst Article 5 of the Biotechnology Directive 98/44EC states that the human body itself is not patentable in Europe,^45^ medical devices are not considered part of the human body at the point of their production. Hence, medical devices would not fall within the Article 5 exclusion at the point of the development or production of such technologies.^46^ Furthermore, the Directive applies only to biotechnological inventions, not to other categories of inventions. Accordingly, even though one could potentially argue that when a device is later implanted into the human body, it becomes ‘part of the body’ at that stage,^47^ this would not affect the patentability of the invention at the grant stage under this provision.

Furthermore, Article 53(a) EPC (and Article 6 of the Biotechnology Directive 98/444EC)—the so-called morality provision—states that inventions will not be patentable if their commercial exploitation is against *ordre public* or morality. However, this provision is often interpreted in a light-touch manner by the European Patent Office (EPO).^48^ There are differing views on the extent to which this provision should be more broadly interpreted to deny patents at the grant stage.^49^ In practice, legal challenges to patentability based on the morality provision are rarely successful. The EPO guidelines for patent examination state that this provision should be used to deny patentability only in ‘rare and extreme’ circumstances, where ‘it is probable that the public in general would regard the invention as so abhorrent that the grant of patent rights would be inconceivable’.^50^ It has not been used to deny patents over medical devices by virtue of these being integrated with the human body per se, or given how patents over such devices could be used including, in ways which they could be used to limit access to such devices (discussed below). Given the high threshold that the EPO applies around this provision, it is unlikely it would apply it to deny patents over medical devices generally.

Alongside the morality provision, Article 53(c) EPC excludes methods of treating humans and animals, including surgical and diagnostic methods and therapies. However, in practice, this provision only excludes from patentability *methods* practised on or in the human body. It expressly does not exclude the patentability of any products used in such contexts, which could include tools used such as medical devices. Nonetheless, where surgical or therapeutic methods are needed for the medical device to function, it could in certain contexts potentially fall under this exclusion.^51^ Thus, a case-by-case assessment of the patent claims is needed in certain contexts.

A patent claim related to a medical device could also take the form of a ‘methods’ claim. Moreover, whilst the exclusion under Article 53(c) is typically construed narrowly,^52^ this exclusion may be relevant to ‘methods’ claims related to medical devices that perform a diagnostic, surgical or therapeutic function within the human body. Thus, whilst a methods claim related to the use of a medical device does not directly fall foul of Article 53(c), however, for example, a device could be excluded from patentability where the claimed method involves a method of operating the device that requires, as a functional step, the surgical or therapeutic treatment of the body.^53^ However, certain patent claims could be drafted to avoid claiming such aspects, thereby potentially avoiding the exclusion under Article 53(c). Moreover, regarding the methods of diagnosis exclusion, based on how this exclusion is applied,^54^ it would likely only exclude a method from patentability where it carries out a complete diagnostic function, not just an interim step.

For connectable medical devices, patents may, in some cases, extend to certain aspects of the software within a device necessary for it to perform a technical function. Under European patent law, Article 52(2)(c) excludes ‘computer programs as such’ from patentability under the EPC. However, this does not exclude all computer programs from patentability. The EPO Board of Appeal, in *IBM (T1173/97)*, held that:

…a computer program claimed by itself is not excluded from patentability if the program, when running on a computer or loaded into a computer, brings about, or is capable of bringing about, a technical effect which goes beyond the “normal” physical interactions between the program (software) and the computer (hardware) on which it is run.^55^

A computer program that does not have a technical function would not be patentable under the EPC. Its patentability is tied to, among other requirements, having a technical effect or contributing to a technical solution. Some computer programs, which form part of a medical device, may have technical and non-technical features, and questions have arisen around whether inventions with a mixture of technical and non-technical features are patentable.^56^ In Case T154/04 *Duns Licensing Associates*, it was held that patent claims could have technical and non-technical elements, but non-technical features of the claim are not considered if they do not resolve the claimed technical problem, and for the invention to be patentable, there must be a technical problem at stake.^57^ A deeper exploration of such EPO decisions is beyond the scope of this article. For the purposes of the arguments here, based on existing EPO decisions, a computer program that forms part of a medical device may be patentable where it contributes to, or has, a technical effect. Technical effects could range from fulfilling or contributing to enabling a technical function or action of the device.

In short, patents are available over various aspects of integrated medical devices, including parts of the device, certain aspects of technical processes and methods through which a device operates, technical processes for the development and production of devices, and in some instances, patents may be available over aspects of computer programs within such devices that have a technical function.

### B. Copyright and integrated medical devices

Copyright protection extends primarily to original works of art, literary, and scientific works and in the EU context, subsists, generally, in the original author during their lifetime and for 70 years after their death.^58^ Copyright also subsists in ‘computer programs’, which are protected as literary works under EU copyright law (Directive 2009/24/EC),^59^ where a ‘computer program’ is defined as including ‘programs in any form, including those which are incorporated into hardware’.^60^ The term ‘computer program’ here could include source code, object code, assembly code,^61^ and other preparatory materials that can be protected by copyright.^62^ Software, firmware, and their source codes have become integral features of connectable integrated medical devices. For example, software programs embedded within such devices often control how medical devices operate.^63^ In the case of connectable medical devices, this can include how such devices interact with the device-user’s body, such as for example, performing prescribing and diagnosing functions. Thus, copyright over the underlying codes required for such applications and programs to operate is a key IP protection applicable in medical device contexts.

In terms of the potential implications that copyright can have for the operation of medical devices—a point discussed in more detail in Section IV—in general terms, copyright prevents unauthorized persons from making copies of the protected work. It also empowers rightsholders to prevent the distribution, reproduction, and unlawful performance or broadcast of the copyright-protected work. Thus, copyright could be used to prevent the making of unauthorized copies of the underlying protected code by third parties and in some cases could be used to prevent modification of this software (if that modification would involve copying of the code). Indeed, many manufacturers of software-embedded devices embed digital locks on the device, which can lock the device if modification of the code (including, for repair or updating purposes) is attempted. Attempts to change copyright-protected code within a device (even, for example, if the intended outcome is to facilitate a change in how the device itself operates) could be a violation of copyright^64^; we discuss such issues in more detail in Section IV.

### C. Other IPRs and integrated medical devices

Copyright and patents are the main focus of this article as these are key IPRs relevant to medical devices. These IPRs can impact the use of medical devices, which in turn impacts device-users, healthcare practitioners, and healthcare systems. However, for the purposes of completeness, several other types of IPRs can apply to medical devices and must be noted, focusing here specifically on design rights, trademarks, and trade secret protection.

First, design rights may be applicable over elements of the external features of a medical device, including lines, contours, shapes, textures, or colours. Article 4 of the Regulation 6/2002 stipulates the conditions that must be fulfilled before a design is protected in the EU.^65^ It must be new, meaning there are no identical designs in the public domain; the design must have an individual character, meaning that a user can distinguish the design from other designs in the public domain.^66^ Under Article 12, a registered design will be protected for 5 years, and it remains renewable for 25 years. Apart from the conditions stipulated in Article 4, designs considered contrary to public policy and principles of morality cannot be protected. Unlike a patent, the protection offered by a design right does not extend to the functional aspect of a design—Article 8 of the Regulation prohibits the registration of designs that are defined by their technical functions. However, design rights could be used as part of rightsholder(s)’ strategies to protect the aesthetic appearance and design of a medical device. Moreover, when combined with other forms of IP protection, including patents, rightsholders could use design rights over elements of medical devices to strengthen their scope of protection and control over how products are manufactured and appear.^67^

Secondly, trademark protection may apply to the brand name and potentially other aspects (where non-traditional trademarks may apply) of a medical device.^68^ Trademark protection could extend to certain aspects relevant to the medical device context, including specific words, signs, images, colours, the shape of goods, packaging, etc.^69^ A trademark is eligible for protection if it is distinctive and therefore capable of distinguishing the goods and/or services of its proprietor from the goods and/or services of other undertakings.^70^ The core function of a trademark is to act as a badge of origin and to secure the distinctiveness of products/services. For example, a brand name of a medical device may be protected by a trademark, which enables the trademark proprietor to prevent third parties from using the mark.

Relatedly, Calboli argues that the protection of non-conventional trademarks (shapes,^71^ colours, texture, etc.) can act as a means for rightsholders to extend their control over health technologies.^72^ Calboli observes that empirical evidence suggests that patients may become accustomed to a particular look or feel of a health-related product and may wish to continue using that particular product or one that looks similar to the originator product, even after patent rights relevant to that product have expired and generic versions are available.^73^ However, trademarks over aspects of the shape, colour, or texture could limit competitors’ ability to provide a similar-looking health technology. Moreover, because there is high demand for such simliar looking products, these other IP protections can enable high prices for originator products to be maintained even after patents expire. Some patients may not be able to afford such prices. Moreover, generic companies may have to consider the potential threat of trademark infringement related to generic products. Thus, Calboli has argued that ‘non-traditional trademarks may delay or altogether block access to these therapies after the patents’ expiration’^74^ and may do so for an extensive period.^75^ When trademarks are overlapped with other IPRs over different elements of the medical device, this solidifies rightsholders’ control over a technology, including a medical device.

Third, and finally, similar to how IPRs can operate in the access to medicines context,^76^ in the medical device context, trade secret protection can also be used alongside other IPRs over key aspects of medical devices, thereby increasing rightsholders’ control. Trade secret protection operates by entities maintaining confidentiality around certain aspects of know-how or information related to, for example, the manufacture or use of, an invention. It is generally used where information or knowledge about, for example, a technique or method needed to produce a technology or invention, would be difficult for others to find out or reverse engineer. In such cases, keeping such information secret can be used to protect this technology from third-party use. In the context of medical devices, for example, trade secret protection could be used to protect certain aspects of how a medical device (or its parts) is manufactured.^77^

### D. Reflection: IPRs and connectable or non-connectable integrated medical devices

In sum, a range of IPRs can apply over different elements of connectable and non-connectable medical devices. This section will now reflect briefly on the potential IPRs that can arise in each category prior to examining the potential health and bioethical implications of how such IPRs can be used over medical devices in Section IV.

First, for integrated non-connectable medical devices, taking a prosthetic device as an example, IPRs applicable in such contexts could include patents over components of the device or over the processes to manufacture or use that device (or its component parts). Moreover, the structural appearance, texture, and colour aspects of a device could potentially be protected through design rights. Trademarks may apply over the brand name of the device or a non-traditional mark over aspects such as the shape of the device. Copyright could apply, for example, over other aspects of the device, such as over the instructions around the use of the device.

Similarly, if we consider IPRs over ‘connectable medical devices’, taking a connectable ‘insulin pump’ device as an example, connectable insulin pumps are increasingly designed to interact directly with continuous glucose monitors (CGMs) and remote controls to create a closed-loop artificial pancreas system, which can monitor blood glucose levels and re-adjust based on the device-user’s needs to maintain healthy glucose levels. The insulin pump is typically connected to a software program, which operates via an external device, such as a mobile phone app.^78^ A connectable insulin pump like this can have both software and hardware components, which could be protected by different types of IPRs. For example, hardware components making up the physical device may be protected by patents, while appearance components may be protected by design rights. Trademarks could apply over the brand or shape, which may distinguish the device from other insulin pumps. Alongside this, certain aspects of the software components of a connectable medical device could be patentable if they bring about a technical effect, and relevant aspects of code underpinning software within the device could be protected by copyright.

IPRs over connectable and non-connectable devices can be used to develop or maintain an income stream for rightsholders, thereby protecting rightsholders’ economic interests. This, in turn, as noted, can act to incentivize (and encourage investment in) the development of certain types of medical device technologies. However, alongside this, as noted, IPRs give considerable discretion to rightsholders over key aspects of how a protected technology or subject-matter is used, accessed, modified (depending on the IPR), and such IPRs can be used by rightsholders in a manner that can have negative impacts on access to and use of such devices, with health and bioethical implications. The potential bioethical and health implications of IPRs over medical device technologies have received limited scrutiny to date which we will now consider.

In making such arguments, this article does not argue that IPRs should not be granted to medical devices. Instead, the key point in the analysis that follows is that the breadth of discretion granted to rightsholders by virtue of IPRs needs deeper and more nuanced consideration in medical device contexts due to how medical devices relate to the human body. This includes consideration – as one of us has argued elsewhere – of the need for licensing conditions over how IPRs over medical devices can be *used* which could limit the potential bioethical and health implications at stake, as far as possible, whilst maintaining the incentivising role of IPRs.^79^

## IV. THE POTENTIAL HEALTH AND BIOETHICAL IMPLICATIONS OF HOW IPRs CAN BE USED OVER INTEGRATED MEDICAL DEVICES

This section examines some of the main health and bioethical implications that can arise around how IPRs are used over (elements of) integrated medical devices, with particular reference to patent and copyright protection. In doing so, it highlights the lack of avenues to directly engage with such health and bioethical implications posed by how IPRs could be used over elements of integrated medical devices in Europe. Instead, once a ‘technology’ is deemed patentable, as noted, that technology is treated as fungible with other technologiesunder patent law.^80^ Put simply, provided a technology or subject-matter is deemed to be patentable or copyrightable, once it is IP protected, IP decision-making systems are often agnostic to the nature of the underlying protected technology or subject-matter and how the use of patents or copyright protection over elements of that technology could impact access to or delivery of health care.^81^

This section argues that IPRs over elements of integrated medical device technologies can have four main *potential* health implications, depending on how rightsholders use such IPRs: (A) impacting—including the potential to hinder—access to medical devices for individual device-users and within health systems; (B) impacting access to repair services and/or the production of repair parts for integrated medical devices; (C) impacting device-users’ ability to modify (or engage third parties to modify) their medical devices to suit their health needs or broader preferences (there are broader safety/ethical considerations here which we acknowledge and discuss below); and (D) impacting, including the potential to impede the development of new or improved integrated medical devices. In each context, where relevant, the analysis will indicate how the use of IPRs has the potential to give rise to bioethical issues.

In making such arguments, the section does not aim to provide a comprehensive analysis of all potential health or bioethical implications that IPRs over integrated medical devices can contribute to. It also does not seek to suggest that IPRs will always have negative implications in the medical device context. This article recognises that IPRs are also an important incentive for the development of medical devices. Furthermore, it is not necessarily the existence of an IPR over medical device components that is at issue; rather, it is often how such rights can be used by rightsholders that can give rise to or exacerbate health/bioethical concerns. Moreover, in some cases, it may be a combination of how different types of IPRs are used over a medical device (together with other factors) that culminates in such access or other health-related implications.

### A. IPRs and potential impacts on access to medical devices

First, as with other types of patentable technologies, patents and other IPRs allow rightsholders the ability to exclude others from using the underlying IP-protected technology. This allows rightsholders a key role in setting the terms of access to the technology, including the price they are willing to offer for the use of the patented technology. For example, rightsholders may refuse to license the patented technology to third parties, which can enable the rightsholder(s) to become the sole provider of the technology, enabling the rightsholder(s) to limit the supplies available. This, in turn, can enable rightsholders to exert a higher price for access to that technology, as creating a market with limited supplies and only one supplier forces the market to bear such costs to gain access.^82^

Such issues are exacerbated by the fact that, as discussed above, a range of different types of IPRs generally apply to connectable and non-connectable medical devices, and such rights are often strategically layered to increase rightsholder(s)’ control and extend the duration of IP protection.^83^ If rightsholders refuse to license a patent to third parties, the technologies needed to develop a specific medical device may become unavailable or in short supply. ^84^ This can have a knock-on effect on access to that device—where the price is significantly high, individuals may be unable to afford it, or public health systems may not be able to provide access due to finite public health budgets.

Various examples of IPRs affecting access to connectable and non-connectable devices illustrate this point. For example, advances in prosthetics give rise to significant potential benefits for device-users, including advances in exoskeletons, which have a range of uses including therapeutic uses and aiding mobility for device-users. Yet, the costs of such devices can be prohibitive in some cases,^85^ and such costs are a key barrier for device-users in accessing or using these devices.^86^ For example, if we consider the bionic BiOM Ankle prosthetic, a device that offers a flexible joint at the ankle,^87^ allowing the device-user more movement than previous prosthetics on the market, the cost of this device has previously been reported as impacting affordability and hence access to the device. In 2013, in the USA, it was suggested at that time, that:

The BiOM ankle, though, still has one big problem: It’s “prohibitively expensive,” Albuquerque says. At about $50,000, it costs up to $40,000 more than a standard prosthesis, and isn’t covered by Medicare, Medicaid, or most insurers. That means most amputees can’t afford it.^88^

Similarly, the first exoskeletons approved by the US FDA were reported as being priced between US$69,000 and 85,000.^89^ Other devices, such as the Esko, which is more akin to a mechanical robot attached to the body, were initially reported as costing upwards of US$100,000 per patient—such devices were generally bought or used by clinics for a group of patients rather than by individual patients.^90^ In the UK, the cost of the Esko in 2017 was reportedly approx. £98,000.^91^ In 2016, it was reported that developments such as lightweight exoskeleton devices, including the Phoenix exoskeleton, aimed to reduce costs to US$40,000 or less per device,^92^ but such prices mean it would still be (likely) unaffordable for many to have a customizable exoskeleton.^93^

Whilst we recognize that IPRs are not the only factor that impact the costs of the device, other factors include the costs of materials needed to make such devices and the development and manufacturing costs, etc. Nonetheless, IPRs, including patents, can be a key relevant factor in this context as they mean rightsholders can gain a potential monopoly right, thereby reducing competition in the market for a device or part, which means the market is likely to bear higher prices if there are no or limited alternatives. IPRs that can apply over elements of such technologies provide rightsholders with significant control over the terms they can set for third parties to access such technologies and enable rightsholders to adopt strategies which potentially block third parties from using or producing the patented technology. Where such technologies relate to integrated medical devices, the use of IPRs in this manner can pose health implications as it can impact, individuals’ and clinics’ ability to access and use prosthetic devices and potentially offer (access to) customizable devices where these are clinically indicated such as for rehabilitation purposes. Lack of access to such technologies can potentially impact users’ quality of life and health, and could have knock-on effects on their broader dignity interests by limiting access to appropriate health services.

There are no mandatory legal provisions in European patent law (at the licensing or grant stage) which specify conditions for how patents over medical device technologies should be licensed to address or mitigate the access-to-health implications arising from how such patents can be used. States can use compulsory licensing mechanisms in patent law to mandate rightsholders to license their patent rights over technologies in certain circumstances.^94^ Many States have avenues under which the State could grant a compulsory license, such as where a voluntary license was unreasonably refused. However, compulsory licensing is not necessarily a solution to such issues, as compulsory licenses must be applied for on a patent-by-patent basis, and must be sought in each national State separately. Additionally, compulsory licenses only apply to patents, not other IPRs that may arise over medical devices.^95^ As medical devices will generally be covered by a range of IPRs, compulsory licenses typically only address one part of the IP hurdles to broadening access. Hence, existing avenues, such as compulsory licenses, are of limited effectiveness. They are also not designed to address the types of issues related to how IPRs could be used over key aspects of integrated medical devices. Given the relatively recent nature of integrated medical device technologies, such issues were likely not envisaged at the time of the drafting of the Paris Convention in 1883, which is the basis for the current compulsory licensing legal framework at a regional level in Europe.^96^

We acknowledge here that there are some avenues outside of patent law at the national level to limit the potential impacts of such IPRs on access to health in such contexts. For example, in some jurisdictions, the grant of a tailored final injunction can be made when infringement against the use of an IP-protected technology is found and where granting a full injunction against the use of the infringing technology is contrary to public interests. For example, in *Edwards Lifesciences LLC v Boston Scientific SCIMED INC*.,^97^ where a transcatheter heart valve (THV), the Sapien 3, produced by Edwards, was found to infringe a patent held by Boston Scientific, an injunction was sought against its use. However, the injunction was tailored so that it did not apply to a cohort of patients who needed access to the Sapien 3, the infringing device, where there was no other suitable device available on the market for these patients. The court held that ordering an immediate injunction against using the valve in the context of patients with no alternative device would have deprived them of needed health care.^98^ Thus, a stay was placed on the injunction for this patient cohort, to permit the ‘continued implantation of the Sapien 3 for a period of 12 months’ in order to facilitate time for the re-training of clinicians to use an alternative device. However, the court gave permission for Edwards ‘to apply to extend the stay if it turns out that the period required for re-training is longer than that’.^99^ Thus, a tailored injunction was used to mitigate against access to health issues arising from the full enforcement of the relevant patent.

The facts of the case starkly illustrate the impact that patent use and enforcement can have on access to health, as without the tailored injunction, enforcing the patent fully could mean an infringing device could no longer be used, regardless of the health context. The exception here was allowed because of the public health impacts for a specific category of patients. However, a tailored injunction is a discretionary equitable remedy (a point discussed below). Had this tailored remedy not been provided and if the injunction had required clinicians to stop using the Sapien 3 for all patients, this could have had significant health consequences for any cohorts of patients who had no alternative devices. Moreover, any use of such an alternative device for such patients would need to be approved, which can take considerable time.^100^ Clinicians and specialist nurses participating in such procedures would require retraining to adopt alternative devices approved for such patients.^101^ Indeed, the court found that:

Expediting such training and proctoring will inevitably disrupt waiting lists. Patients waiting to be treated by clinicians who have to be diverted into training will suffer. So too will patients who would otherwise be treated by the proctor.^102^

The potential impacts of enforcing the patent in this context on clinical autonomy is also alluded to in the judgment, at para 58; the court stated:

He [Prof Brecker] is in little doubt that individual operators [clinicians etc] will feel uncomfortable at not being able to use a device that they have become familiar with and may feel less confident at first in treating patients with a new device. Dr Rawlins (who is already familiar with the Sapien 3) considers that it will take approximately 50 implantations to become fully familiar with the Evolut R and to maximise clinical outcomes using it.

Here, we see clear illustrations of how general patent enforcement could impact both access to and delivery of health care in certain medical device contexts, with such issues being averted in this case via tailoring the injunction on the basis of public health issues at stake. However, injunctions are equitable remedies granted on a case-by-case basis and whether injunctions can be tailored – whereby typically damages will be awarded in lieu in certain contexts – is also considered on a case-by-case basis, with high thresholds applying. Such solutions are often time-limited and are not designed as a general avenue to address the potential impact of patents (and other IPRs) over access to medical devices. Furthermore, in some instances, it is at least plausible that the threat of patent infringement litigation may lead a medical device provider to halt the production and provision of the alleged infringing device, given the high costs of patent litigation. Where this happens, a challenge like the one in the *Edwards case* may not come before a court; parties may settle in advance of litigation going to court, and as part of such settlements in many cases the alleged infringer would agree to stop using the alleged infringing technology. In such instances, device-users may be deprived of a medical device that may be necessary for their health needs, and this could have serious consequences.

### *B.* Repairing IP-protected medical devices: Potential impacts of IPRs on Repair and Bioethical Implications 

Secondly, the hardware components of connectable and non-connectable devices may need replacement or repair. However, as medical device parts are typically protected by a range of IPRs, including patents, the production of a new part to replace an old part of a medical device could constitute patent infringement if it was found to constitute the act of ‘making that product’.^103^ In determining whether making a new part infringes a patent, much will depend on how integral a part is to the patented technology in question.^104^ If a third party were to make such parts (without a license from the rightsholder) that are deemed essential to the patented technology, even if they were making such parts for the purposes of repairing an existing device, they could be infringing the patent. Hence, rightsholders not only control making of a device itself, they can also have some control over the supply or making of repair parts for medical devices in certain contexts.^105^ In practice, commerical services for the maintenance/repair of such devices may only be legally possible by rightsholders (or their licensees), which can lead to high costs and other limitations on access to such repair/maintenance services, as discussed below.

Furthermore, in the connectable device context, the ongoing control exercised by rightsholders over devices, which is impacted by a range of factors including IPRs, has the potential to directly impact device users’ ability to use existing devices in certain contexts. This is illustrated by considering, for example, the case of the Second Sight Argus bionic eye,^106^ where over 350 people had Second Sight’s Argus bionic eye medical devices implanted into their body, allowing them to regain a form of ‘vision’.^107^ To have these devices implanted, patients were required to undergo surgical procedures to fit these devices within their bodies. In 2020, Second Sight, the company that manufactured the devices ran into financial difficulties, and some device-users reported their devices stopped working or started malfunctioning. Such device-users also reported that they were no longer able to obtain device upgrades at that time as these were not being produced by the company.^108^ This effectively meant device-users lost the form of ‘vision’ they had regained since having the technology implanted, causing significant distress.^109^

As one of us has discussed elsewhere,^110^ the Second Sight issues were not caused by IPRs; rather, such issues arose largely due to financial issues of the company, and a key difficulty here for device-users was the lack of ongoing legal obligations on a manufacturer to continue to provide upgrades and services for this medical device. However, IPRs could, at least in theory, also be used in a manner that could lead to a simliar scenario arising.^111^ For example, a rightsholder of a patentable component of a medical device may decide they no longer wish to produce software upgrades for an existing medical device; however, they would still retain IPRs over that medical device (or elements of this). Rightsholders could refuse to license third parties to produce such upgrades for the underlying code embedded in the device to enable it to operate. Moreover, changing or copying this code, which may be needed to upgrade it, could constitute copyright infringement if undertaken by third parties. Thus, device-users may be left with no upgrades being provided by the rightsholder, and third parties may be unable to provide upgrades due to IPRs and other factors. Indeed, it may be in the financial interests of rightsholders to adopt such strategies that enable them to block existing device updgrades or repairs, thereby creating a need or market for new devices.^112^ The result could be that a device-user loses the use of the original device and must change devices, which may impose additional costs and, in some cases, these upgrades or new devices may be inaccessible to the device-user. Furthermore, changing a device may not be the preference of the device-user if they have become familiar with the existing medical device and trust that device.^113^ In such cases, IPRs could be used to impact users’ autonomy over which device or use they can obtain, alongside having a practical effect on their access to healthcare more generally.

Despite such implications, no tailored provisions in European patent law require rightsholders to provide ongoing maintenance or repairs of patent-protected hardware components or processes for medical devices. There are also no mandatory copyright provisions that require rightsholders to enable (or at least not restrict) third parties to upgrade codes within medical devices where this is required for existing devices to function (even when rightsholders do not provide this themselves).^114^ Whilst this issue could be dealt with by other areas of law, such as via regulatory conditions on the approval of a technology for use, contractual requirements, or legislative provisions, currently, such issues are in many cases left at the voluntary discretion of rightsholders or licensees given (amongst other factors) the discretion given to them by virtue of how IPRs operate.^115^

### C. Impacts of IPRs on the modification of medical devices

Thirdly, IPRs can be used in a way that impacts the extent to which, if any, device-users can modify (or have modified by third parties) existing devices.^116^ Device-users may wish to modify their devices for a range of reasons. For example, the first commercially produced hybrid-closed-loop artificial pancreas systems (used to manage certain types of diabetes) to monitor and provide automated insulin were not released until 2016 in the USA (MiniMed 670G (Medtronic).^117^ Prior to this, diabetes patients and their families campaigned for closed-loop systems. Under the #WeAreNotWaiting campaign, members of the diabetes community sought to make DIY closed-loop or artificial pancreas systems for use.^118^ Groups such as OpenAPS launched a website in February 2015 that provided information for Type 1 diabetes patients on closed-loop systems, including instructions on how to make DIY systems.^119^ There are safety and ethical issues around device-users making their own medical devices or altering existing medical devices—as users’ modifications could pose health risks. However, there are also an increasing number of groups seeking to modify their personal medical devices or have these devices modified by third parties for a range of reasons.^120^

Such individuals or groups can face challenges around modifying existing medical devices, including challenges posed by IPRs. Where IPRs, including patents, apply over hardware and software components of a device or copyright over the source code, a third party that modifies such devices for sale or distribution to the public could be infringing applicable patents or copyright protection. Accordingly, IPRs could be used to limit the ability of third parties to modify devices or tools to achieve this for sale. Moreover, the threat of IP infringement litigation may deter such actions.

A medical device-user could, in theory, modify their medical device for personal use and not infringe applicable patents, as under many national patent laws, acts done for personal and non-commercial use typically would not constitute patent infringement.^121^ However, medical device-users may be unable to modify their own devices, given the technical skills that would likely be needed, or may be concerned about the risks of doing so. Instead, they may want to request a third-party provider to modify the device to their requirements. However, unless rightsholders have licensing agreements in place for such purposes with that third party, actions that modify patented components of a medical device for commercial purposes by a third party could amount to patent infringement (or an infringment of other IPRs applicable).^122^

For example, to modify how a connectable device, such as an artificial pancreas system, operates, one may need to copy or change the source code. If that source code is protected by copyright, changes to this code could be an infringement of the applicable copyright. To date, such IP avenues do not appear to have been pursued by rightsholders.^123^ However, this does not mean such IPRs will not be enforced in this way in future.

Furthermore, rightsholders may use technological restrictions on a medical device that prevents it from being modified, and IPRs can be used to maintain such restrictions. For example, in the USA, some of the initial artificial DIY pancreas closed-loop systems were reportedly only possible due to a security flaw in an older Medtronic insulin pump produced before 2015, which allowed users to hack the pump and connect it with the CGM.^124^ That flaw was subsequently addressed by the manufacturer. Thus, people who wished to set up or use DIY systems needed to procure an older insulin pump with this flaw.^125^ Regardless of why device-users wished to alter such devices, generally (under relevant IP laws), they do not have legal recourse to force manufacturers to allow them or third parties to make devices modifiable. Instead, rightsholders can argue that technical fixes that block third parties’ ability to modify devices are needed to protect underlying copyright protections, and attempts to change these, including tampering with the technical fixes, could violate IPRs. Hence, IPRs can be used to bolster technological avenues that stop users and third parties from modifying devices.^126^

Here, we concede that rightsholders may have valid concerns about medical devices being modified by device-users or third parties on the device users’ behalf. Given the potential health and safety implications, liability issues or uncertainties may arise in the event of risks materializing as a result of modifications,^127^ and modifications may also be contrary to medical device regulations. Moreover, relatedly, IPRs are not the only legal instrument or sole factor impacting freedom of third-party modification. The broader issues around IPRs and repair of medical devices, are beyond the scope of this article.^128^ Our key point is that, given how IPRs operate, the current system allows rightsholders to use various types of IPRs in a way that extends their control not just over the sale of the device but also (especially for connectable devices) in a way that could impact the modification of devices. In short, depending on how such rightsholders use relevant IPRs, their choices can restrict device-users’ ability to modify or repair such medical devices.^129^ Here, we acknowledge that the use of IPRs in this way aligns with how IPRs, including patents, ordinarily operate in other technological contexts. However, given the nature of the underlying patented technology—the integrated medical device—and its relationship with how the human body functions, our core argument is that such implications around the use of IPRs over such technologies warrant deeper consideration and recognition within the health law and bioethics contexts.

Importantly, we are not necessarily suggesting here that DIY modification of medical devices should be facilitated; this normative issue is beyond the scope of this article. Instead, our point is that IPRs are one factor that can impact the extent to which third parties can legally modify medical devices and the extent to which users can have their devices modified by entities that are not the rightsholders. This could impact the device-user’s autonomy over their health-care options, as well as their human dignity, if the device they have is not working as effectively as could be possible without such modification. To date, there has been limited scrutiny of the impacts of such IPRs on device-users’ experiences. We argue that such IPR issues warrant greater nuanced consideration—alongside the broader legal and regulatory questions which arise here—from the health law and bioethics communities.

### D. IPRs and the development of integrated medical devices

Finally, given the multiple types of IPRs that can apply over medical devices, such rights can both encourage but also limit the ability of manufacturers and researchers to produce new devices or develop existing devices. This issue is not unique to medical devices; it stems from the double-edged nature of IPRs, which, on the one hand, can be used to incentivize new technological developments, for example, in the case of patents, by providing rightsholders with the ability to stop others from using their invention for the duration of patent right. On the other hand, the exclusive nature of IPRs means that third parties who need to use, for example, a patent-protected technology as part of an intended new technology, are unable to do so without a license from the original rightsholder, who may refuse this.^130^ Complex layers of IPRs over existing technologies can arise, which makes this issue more difficult.^131^ Thus, existing patents and other IPRs over elements of a medical device can be used in a manner that blocks innovation. Third parties may have to change proposed devices or invent around existing patent-protected technologies. Rightsholders may try to ‘evergreen’ patent rights, i.e., seeking to develop minor changes on an existing part of a medical device technology, to apply for new patents on this and extend their patent protection.^132^ Moreover, by combining patent, trade secret, copyright, and trademark protections over different aspects of devices, the breadth and length of control that rightsholders have can be strategically extended beyond, for example, an initial 20-year patent grant.^133^ As in other fields, the role of IPRs can be used to block competitor products and processes, which is indeed part of how IPRs operate. A nuanced examination of the grant and use of patents (and use of other IPRs) is needed, as without any IPRs certain types of innovation may be disincentivized. However, certain uses of IPRs over elements of medical device technologies, also has the potential to hinder the development of new devices (or to hinder developments on existing devices).

## V. CONCLUDING REFLECTIONS

This article has demonstrated that IPRs over integrated medical devices, and how rightsholders can use such rights, may impact how device-users can access, use, repair, and modify such devices; it can also limit how such devices can be developed, used and modified by third parties. This may give rise to health-related implications. For instance, depending on how IPRs are implemented and enforced, it may lead to high costs for a device, leading to implications for access; it may also constrain the development of new health technologies and even impact the ability to modify (including repair) existing medical devices.^134^ In public health systems, where IPRs over health technologies are used in a way that leads to high costs, this can lead to difficulties for public health systems in providing access to such devices for all individuals who clinically need them. It may exacerbate healthcare rationing issues, and may impact the availability of suitable devices (if any) for potential device-users, and for clinical care. Accordingly, IPRs can be used to constrain patients’ and healthcare systems’ use and accessibility of medical devices, with knock-on implications for bioethical interests.

Having said this, IPRs also play a role in incentivizing the development of medical device technology. They can be used to provide an income stream for rightsholders to recoup the costs associated with developing these technologies. Within the current health innovation model, this is often a key consideration for certain actors, particularly companies, around their investments in technologies for research and development purposes. Here, we are not arguing that this is the only (or most efficient) viable health innovation model, such issues are beyond the scope of this article. Instead, our point is that, aside from the economic function of patents, the discretion given to rightsholders (by virtue of how patents and other IPRs operate) over the protected technology can can pose considerable health and bioethical implications. We acknowledge here that it is not the existence of IPRs over medical devices *per se* that leads to potential health implications; instead, it is often how such IPRs can be used over IP protected technologies, which typically does not distinguish between whether the technology in question relates to a medical device or other context.

There are limited avenues to directly engage with the health and bioethical implications of how IPRs can be used over integrated medical devices under existing IP systems, and there has been limited examination of these issues for medical devices. This article has sought to fill this gap and to argue that deeper consideration is needed around the breadth (and limits) of rightsholders’ discretion over how they use IPRs related to medical devices, given the health and bioethical implications at stake. This could be achieved by licensing restrictions or principles that enable rightsholders to obtain some of the benefits of patent (or other IP protection) but limit, in certain contexts, how such patents (and other IPRs) can be used to address potential bioethical issues that can arise. The purpose of this article is not to develop what such licensing principles would look like; instead, it has aimed to elucidate, as a first step, the need for greater scrutiny over rightsholders’ discretion around how they use IPRs—particularly patents—over integrated medical devices. In doing so, our core argument is that greater interdisciplinary scrutiny is vital around the discretion given to rightsholders over how they use IPRs related to integrated medical devices, given the potential health and bioethical implications at stake.

## Footnotes

1 art 5, Biotechnology Directive 98/44EC.

2 See Aisling McMahon, ‘Patents over Technologies Related to How We Treat, Use and Modify the Human Body: An Urgent Need for Greater Bioethics Scrutiny’ (Working Paper 2024, on file with author).

3 As will be discussed below, the use of the term ‘integrated’ medical device draws on Quigley and Ayihongbe’ conception and use of the term in: Muireann Quigley and Semande Ayihongbe, ‘Everyday Cyborgs: On Integrated Persons and Integrated Goods’ (2018) 26 Medical Law Review 276; On the broader legal challenges posed by ‘integrated medical devices’ in the ‘everyday cyborg’ context, see: “Everyday cyborgs 2.0 - Law’s boundary work and alternative legal futures” Wellcome Trust funded project (Principal Investigator: Prof Muireann Quigely, University of Birmingham) https://blog.bham.ac.uk/everydaycyborgs/, accessed 4 February 2025. For a discussion of ‘everyday cyborgs’ more generally, see: Gill Haddow and others, ‘Cyborgs in the Everyday: Masculinity and Biosensing Prostate Cancer’ (2015) 24 Science as Culture 484; Gill Haddow, *Embodiment and Everyday Cyborgs: Technologies that Alter Subjectivity* (Manchester University Press 2021).

4 See Quigley and Aydihongbe (n 3). See also: McMahon (n 2). The broader potential bioethical implications of patents over technologies related to the human body, are explored in detail by the ERC PatentsInHumans Project No. 101042147), led by Aisling McMahon. Medical devices are one of the case studies within the project. For further information, see www.patentsinhumans.eu

5 See also Quigley and Ayihongbe (n 3) 299–301, which highlights the lack of clarity around key aspects of IPRs and medical devices, and questions that arise around ‘…the appropriateness of third-party control over devices, which, in essence, become integrated with persons’ (p 300).

6 A 2020 WTO/WHO/WIPO study on access to medical technologies noted that: ‘Little published research is available on the issue of access to medical devices’, see World Trade Organization, World Health Organization and World Intellectual Property Organisation, *Promoting Access to Medical Technologies and Innovation Intersections between Public Health, Intellectual Property and Trade*, 2nd edn (World Trade Organization 2020) 227. Existing literature on IPRs and medical devices, includes: Quigley and Ayihongbe (n 3) 299–302; Abbe Brown and others, ‘Body Extension and the Law: Medical Devices, Intellectual Property, Prosthetics and Marginalisation (Again)’ (2018) 10 Law, Innovation and Technology 161; Hembadoon I Oguanobi, ‘Broadening the Conversation on the TRIPS agreement: Access to Medicines Includes Addressing Access to Medical Devices’ (2018) 21 Journal of World Intellectual Property Law 70; Richard Gold and others, ‘Are Patents Impeding Medical Care and Innovation?’ (2009) 7 PLoS Medicine 1, 2; Fariborz Moazzam and Michael D Bednarek. ‘Intellectual Property Protection for Medical Devices’ in Karen Becker and John J Whyte (eds), *Clinical Evaluation of Medical Devices: Principles and Case Studies* (Humana Press 2006) 117–37.

7 In doing so it focuses primarily on the EU context, and with reference to patent law, it will focus on ‘European’ patent law, discussed further below. However, here, it can be noted, that by ‘European’ patent law the article refers to the framework applicable under the European Patent Convention 1963, as amended. For a discussion, see Aisling McMahon, ‘An Institutional Examination of the Implications of the Unitary Patent Package for the Morality Provisions: A Fragmented Future Too Far?’ (2017) 48 International Review of Intellectual Property and Competition Law 42; Aisling McMahon, ‘Decision-Makers, Institutional Influences and the Role of Ethical issues in the Patenting of Biotechnological Inventions in Europe: Enter the Unitary Patent System’ in Duncan Matthews and Paul Torremans (eds), *European Patent Law: The Unified Patent Court and the European Patent Convention* (De Gruyter 2023) 517–532; Karen Walsh, *Fragmentation and the European Patent System* (Hart/Bloomsbury 2022) ch 1.

8 For an overview/critique of these arguments in the vaccine context, see Mark Eccleston-Turner, ‘The Economic Theory of Patent Protection and Pandemic Influenza Vaccines: Do Patents Really Incentivise Innovation in the Field’ (2016) 42 American Journal of Law & Medicines 577. See also: Giovanni Dosi and others, ‘Do Patents Really Foster Innovation in the Pharmaceutical Sector? Results from an Evolutionary, Agent-based Model’ (2023) 212 Journal of Economic Behaviour and Organisation, 564.

9 M Scherer, ‘Pharmaceutical Innovation’ in Bronwyn H Hall and Nathan Rosenberg (eds), *Handbook of the Economics of Innovation (Volume 1)* (Elsevier 2010) 539–74.

10 As will be discussed below, this is an exclusive right as once a patent is granted third parties must seek permission from the rightsholders (often via a license exchanged for monetary value) and hence, rightsholders have exclusive rights over the patented technology.

11 Scherer (n 9).

12 For a discussion and critique of the role of patents as incentives in a range of contexts, see Mark A Lemley and Dan L Burk, ‘Policy Levers in Patent Law’ (2003) 89 Virginia Law Review 1575, Minnesota Public Law Research Paper No. 03-11, UC Berkeley Public Law Research Paper No. 135 <http://dx.doi.org/10.2139/ssrn.431360> accessed 4 February 2025. In the COVID-19 context, see discussion in Siva Thambisetty and others, ‘The TRIPS Intellectual Property Waiver Proposal: Creating the Right Incentives in Patent Law and Politics to End the COVID-19 Pandemic’ (24 May 2021) LSE Legal Studies Working Paper No. 06/2021 <https://ssrn.com/abstract=3851737> acessed 4 February 2025; Siva Thambisetty and others, ‘Addressing Vaccine Inequity during the COVID-19 Pandemic: The TRIPS Intellectual Property Waiver Proposal and Beyond’ (2022) 81 The Cambridge Law Journal 384.

13 For example, see discussion in: Cindy Bors and others, ‘Improving Access to Medicines in Low-Income Countries: A Review of Mechanisms’ (2015) Journal of World Intellectual Property Law 1–28.

14 Eg, see Brigitte Tenni and others, ‘What is the Impact of Intellectual Property Rules on Access to Medicines? A Systematic Review’ (2022) 18 Global Health 34; Karen Walsh and others, ‘Intellectual Property Rights and Access in Crisis’ (2021) 52 International Review of Intellectual Property and Competition Law 379; Duncan Matthews, ‘The Right to Health and Patents’ in Christophe Geiger (ed), *Research Handbook on Human Rights and Intellectual Property* (Edward Elgar Publishing 2015) 496; Reed F Beall and others, ‘Is Patent “Evergreening” Restricting Access to Medicine/Device Combination Product?’ (2016) 11 PLoS One 1.

15 For eg, Kristen Nugent, ‘Patenting Medical Devices: The Economic Implications of Ethically Motivated Reform’ (2008) 17 Annals of Health Law 135; Richard E Gold and others, ‘Are Patents Impeding Medical Care and Innovation?’ (2010) 7 PLoS Medicine 1; Muhammad Z Abbas, ‘Patent Law and 3D Printing Applications in Response to COVID-19: Exceptions to Inventor Rights’ (2022) 25 Journal of World Intellectual Property 319; Brown and others (n 6); Quigley and Ayihongbe (n 3); Tiffany E Chao and Gita N Mody, ‘The Impact of Intellectual Property Regulation on Global Medical Technology Innovation’ (2015) 1 BMJ Innovations 49.

16 See Aisling McMahon, ‘Global Equitable Access to Vaccines, Treatments and Diagnostics for Covid-19: The Role of Patents as Private Governance’ (2021) 47 Journal of Medical Ethics 142.

17 Abbas (n 15).

18 Dorothy R Auth, ‘COVID-19 Update: Patent Rights in the COVID-19 Pandemic: How will Industries and Governments Respond?’ National Law Review <https://www.natlawreview.com/article/covid-19-update-patent-rights-covid-19-pandemic-how-will-industries-and-governments> accessed 20 December 2023.

19 McMahon (n 16); see also McMahon (n 2).

20 Jefrey K Aronson, Carl Heneghan and Robin E Ferner, ‘Medical Devices: Definition, Classification, and Regulatory Implications’ (2020) 43 Drug Safety 84; Josep Malvehy and others, ‘New Regulation of Medical Devices in the EU: Impact in Dermatology’ (2022) 36 Journal of the European Academy of Dermatology and Venereology 360; Fiona Masterson and Kathryn Cormican, ‘Overview of the Regulation of Medical Devices and Drugs in the European Union and the United States’ (2013) 47 Therapeutic Innovation & Regulatory Science 715; Filippo Pesapane and others, ‘Artificial Intelligence as a Medical Device in Radiology: Ethical and Regulatory Issues in Europe and the United States’ (2018) 9 Insights Imaging 745, 748.

21 Regulation (EU) 2017/745 of the European Parliament and of the Council of 5 April 2017 on medical devices [2017] OJ L 117/1 (art 2(1)); See also Directive 93/42/EEC (medical devices) (art 1); Directive 2007/47/EC, art 1. A separate definition is provided for in vitro diagnostic medical devices – art 2(2) of Regulation 2017/746.

22 See Brown and others (n 6).

23 See World Health Organization, ‘Medical Devices’ <https://www.who.int/teams/health-product-policy-and-standards/assistive-and-medical-technology/medical-devices> accessed 20 December 2023; For a detailed definition of ‘medical device’, the WHO referred to the Global Harmonization Task Force Study Group 1, ‘Definition of Terms “Medical Device” and “In Vitro Diagnostic (IVD) Medical Device”’ The Global Harmonisation Task Force, 16 May 2012, para 5.1.

24 Aronson, Heneghan and Ferner (n 20).

25 Marco Racchi and others, ‘Insights into the Definition of Terms in European Medical Device Regulation’ (2016) 13 Expert Review of Medical Devices 907.

26 See discussion in Timo Minssen, Marc Mimler and Vivian Mak, “When Does Stand-Alone Software Qualify as a Medical Device in the European Union?—The CJEU’s Decision in Snitem and What It Implies for the Next Generation of Medical Devices” (2020) 28 Medical Law Review 620; Laura Downey and Muireann Quigley, ‘Software as a Medical Device: A Bad Regulatory Fit?’ (*Everyday Cyborgs Blog*, March 2021) <https://blog.bham.ac.uk/everydaycyborgs/2021/03/15/software-as-a-medical-device-a-bad-regulatory-fit/> accessed 11 December 2023; Kasper Ludvigsen, Shishir Nagaraja and Angela Daly, “When is Software a Medical Device? Understanding and Determining the “Intention” and Requirements for Software as a Medical Device in European Union Law” (2022) 13 European Journal of Risk Regulation 78.

27 The classification of 3D bio-printed tissues depends on a range of factors; see European Parliamentary Research Services, 3D Bio-printing for Medical and Enhancement Purposes: Legal and Ethical Aspects’ (July 2018) PE 614.571 <https://www.europarl.europa.eu/RegData/etudes/IDAN/2018/614571/EPRS_IDA(2018)614571(ANN2)_EN.pdf> accessed 20 December 2023; Tajanka Mladenovska, ‘The Regulatory Challenge of 3D Bioprinting’ (2023) 18 Regenerative Medicine, 659; Anotonia Cronin, Rebecca Thom and VANGUARD Consortium, ‘Regulatory Challenges at the Intersection of Cellular and Medical Device Therapies in Europe: The Case of the Bioartificial Pancreas’ (2023) 5 Law, Technology and Humans 114.

28 Racchi and others (n 25) 907

29 Regulation (EU) 2017/745 of the European Parliament and of the Council of 5 April 2017 on medical devices [2017] OJ L 117/1. Here, we acknowledge that not all medical devices have therapeutic functions.

30 Global Harmonization Task Force Study Group 1 (n 23).

31 See discussion in Minssen, Mimler and Mak (n 26).

32 Quigley and Ayihongbe (n 3) who state: “...everyday cyborgs are integrated persons; that is, the integration of the biological person with the technological.” 277–278. Moreover, they conceptualize everyday cyborgs as follows: ‘For us, everyday cyborgs are (i) persons with ‘technologies of the carpentry kind’ such as artificial joints, as well as simple prosthetics such as aesthetic arm prostheses and cosmetic ocular prosthetics (artificial eye); (ii) those carrying devices, such as insulin pumps, which are automated to monitor blood glucose and deliver insulin; (iii) persons with implanted medical devices such as pacemakers, ICDs, cochlear implants, or deep brain neurostimulators, which regulate or replace some physiological function or other; and (iv) persons with complex prosthetics such as retinal prostheses (‘the bionic eye’) or myoelectric prosthetic arms.’ Quigley and Ayihongbe (n 3) 291. See also Gill Haddow and others, ‘Cyborgs in the Everyday: Masculinity and Biosensing Prostate Cancer’ (2015) 24 Science as Culture 484; Haddow and others (n 3). For a discussion of tangible rights and the human body, which is outside the scope of this article, see: Muireann Quigley, Self-ownership, Property Rights, and the Human Body: A Legal and Philosophical Analysis (CUP, 2018).

33 For a detailed argument focusing on a range of technologies related to the human body McMahon (n 2).

34 McMahon (n 2).

35 For biotechnologies, Dickenson states: ‘…modern biotechnology muddies the clear distinction between things external to our bodily selves and those intrinsic to us’. Donna Dickenson, *Property in the Body: Feminist Perspective*, 2nd edn (CUP 2017) 29. For a discussion that resonates in the tangible property in the body context, see Muireann Quigley, ‘Property in Human Biomaterials—Separating Persons and Things?’ (2012) 32 Oxford Journal of Legal Studies 659; See also: Quigley and Aydihonbe (n 3).

36 Bartjan Maat and others, ‘Passive Prosthetic Hands and Tools: A literature Review’ (2018) 42 Prosthetics and Orthotics International 66.

37 For a discussion of the ownership of tangible elements of medical devices, and how this differs from (and may not align in many cases) with intangible rights over elements of a device, see Quigley and Ayihongbe (n 3).

38 TRIPS Agreement, Marrakesh Agreement Establishing the World Trade Organization, Annex 1C, 33 I.L.M. 81 (1994), arts 27 and 28.

39 It should also be noted that most recently a unitary patent system for participating EU States has commenced, and where relevant aspects of this system will also be referred to; however, the European Patent Organization remains the main European regional grant body for patents under this system. See discussion of complex institutional framework with Europe for patent law in Walsh (n 7); McMahon (n 7); Aisling McMahon ‘Decision-Makers, Institutional Influences and the Role of Ethical issues in the Patenting of Biotechnological Inventions in Europe: Enter the Unitary Patent System’ in Paul Torremans and Duncan Matthews (eds), *European Patent Law: The Unified Patent Court and the European Patent Convention.* (De Gruyter 2023) 517–532; Aisling McMahon, ‘Institutions, Interpretive Communities and Legacy in Decision-Making’ in Niamh NicShuibhne and Edward Dove (eds), *Law and Legacy in Medical Jurisprudence: Essays in Honour of Graeme Laurie* (CUP 2022) 345–366.

40 art 52, European Patent Convention (EPC) [1973], as amended.

41 art 53, European Patent Convention 1973, as amended.

42 art 27(3), TRIPS Agreement.

43 On the fungibility of patentable technologies, see TT Arvind and Aisling McMahon, ‘Commodification, Control, and the Contractualisation of the Human Body’ in Elodie Bertrand, Marie-Xavière Catto and Alicia Mornington (eds), *The Limits of the Market: Commodification of Nature and Body* (Mare&Martin 2020) 43–68.

44 ibid.

45 The Directive is supplementary interpretation for the EPC in the biotechnology context.

46 The broader bioethical issues posed by how patents (and other IPRs) may apply over a range of technologeis related to the human body, are discussed in McMahon (n 2).

47 ibid. For a broader discussion around the integration of bodies and goods (devices) in this context and the legal challenges posed, see Quigley and Aydihongbe (n 3).

48 See McMahon (n 7); Aisling McMahon, ‘The Morality Provisions in the European Patent System: An Institutional Examination’ (PhD thesis, University of Edinburgh 2016).

49 See Karen Walsh and Naomi Hawkins, ‘Expanding the Role of Morality and Public Policy in European Patent Law’ in Paul Torremans (ed), *Intellectual Property Law and Human Rights* (Wolters Kluwer 2020) ch 27, 869–906; Justine Pila, ‘Adapting the Ordre Public and Morality Exclusion of European Patent Law to Accommodate Emerging Technologies’ (2020) 38 Nature Biotechnology 555; Ana Nordberg, ‘Patents, Morality and Biomedical Innovation in Europe: Historical Overview, Current Debates on Stem Cells, Gene Editing and AI, and de lege ferenda Reflections’ in Daniel Gervais (ed), *Fairness, Morality and Ordre Public* (Edward Elgar, 2020); Oliver Mills, *Biotechnological Inventions: Moral Restraints and Patent Law* (Routledge 2010) chs 1 and 4.

50 European Patent Office, Guidelines for Examination, Part G, chs 2 and 4.1 <https://www.epo.org/en/legal/guidelines-epc/2023/g_ii_4_1.html> accessed 20 December 2023.

51 EPO Board of Appeal, Case T1731/12; See earlier decision in Case T 0775/97 of 03 April 2001. These decisions are discussed in Thorsten Bausch, ‘Functionally Defined Medical Devices at the EPO—Is This a Thing of the Past?’ (*Kluwer Patent Blog*, 28 September 2019). <https://patentblog.kluweriplaw.com/2019/09/28/functionally-defined-medical-devices-at-the-epo-is-this-a-thing-of-the-past/> accessed 28 October 2024.

52 See discussion in Sigrid Sterck and Julian Cockbain, *Exclusions from Patentability: How Far Has the European Patent Office Eroded Boundaries?* (CUP 2012) ch 5.

53 T 0082/93 (Cardiac pacing) of 15 May 1995.

54 See discussion of this provision in Sterck and Cockbain, (n 52); see also Abbe Brown and others, *Contemporary Issues in Intellectual Property Law* (OUP 2022).

55 T 1173/97 (Computer Program Product) 01-07-1998, para 13 <https://www.epo.org/en/boards-of-appeal/decisions/t971173ep1> accessed 20 December 2023. The line of cases leading to the decision in IBM include Vicom—T 208/84 (Computer-related invention), 15 July 1986; and T 26/86 (X-ray apparatus) Koch & Sterzel, 21 May 1987; see discussion in David Pearce, ‘Patentability of Software in Europe’ in Hayleigh Bosher and Eleonora Rosati (eds), *Developments and Directions in Intellectual Property Law: 20 Years of The IPKat* (OUP 2023) 486.

56 This includes questions around how this should factor into an assessment of the inventive step criteria as part of the patentability test, see T 641/00 (*COMVIK*). T 258/03 (Auction method/Hitachi) 21 April 2004 discussed in: Pearce (n 55) 486. See also T 0931/95 Pension Benefits System, para 2.

57 Case T154/04 DUNS Licensing Associates 15 November 2006; See earlier cases T 641/00 (*COMVIK*); T 258/03 (Auction method/Hitachi) 21 April 2004; See Pearce (n 55) 488

58 art 1, Directive 2006/116/EC of the European Parliament and of the Council on the term of protection of copyright and certain related rights of 12 December 2006. The minimum term of protection is 50 years after death, as set out under Art 7, Berne Convention on the Protection of Literary and Artistic Works of September 9 1886, as amended. However, as in EU context, States/regions can provide a longer term.

59 Directive 2009/24/EC, Recital 6 and art 1(1), which stipulates that ‘Member States shall protect computer programs, by copyright as literary works within the meaning of the Berne Convention for the Protection of Literary and Artistic Works.’ In the UK context, see *Gates v Swift* [1982] 339; *Sega Enterprises v Richards* [1983] FSR 73; *Thrustcode v WW Computing* [1983] FSR 503, as discussed in: Lionel Bently and others, *Intellectual Property Law,* 6th edn (OUP 2022) 71.

60 ibid, Recital 7.

61 ibid 71.

62 Directive 2009/24/EC, art 1.

63 For a discussion of relevance of copyright to operation of medical devices such as ventilators in the COVID-19 context, see: Sean Flynn, Erica Nkrumah and Luca Schirru, ‘International Copyright Flexibilities for Prevention, Treatment and Containment of COVID-19’ (2022) 29 The African Journal of Information and Communication 1, 6.

64 For a discussion of how this could impact repair/updating of electronic devices more generally, see: Sahra Svensson and others, ‘The Emerging “Right to Repair” legislation in the EU and the U.S’ in *Going Green Care Innovation 2018*, Vienna, Austria, 2018 <https://lucris.lub.lu.se/ws/portalfiles/portal/63585584/Svensson_et_al._Going_Green_CARE_INNOVATION_2018_PREPRINT.pdf> accessed 20 December 2023; see Aaron Perzanowski, *The Right to Repair: Reclaiming the Things We Own* (CUP 2022) 9, 111–24.

65 Council Regulation (EC) No 2002/6 of 12 December 2001 on Community Designs [2002] OJ L 003). The EU recently adopted a new design rights legal reform package which includes: the Regulation (EU) 2024/2822 of the European Parliament and of the Council of 23 October 2024 amending Council Regulation (EC) No 6/2002 on Community designs and repealing Commission Regulation (EC) No 2246/2002. This Regulation will amend Regulation 6/2002, many of its provisions within will come into effect on 1 May 2025 (with other provisions coming into effect in 2026). Also of relevance, is the new Directive (EU) 2024/2823 of the European Parliament and of the Council of 23 October 2024 on the legal protection of designs (recast). This Directive entered into force in December 2024, and national EU States have 36 months to transpose this into national law. These new laws will amend the design rights framework under EU law. Of particular note, is Art 19 of the Directive which includes a ‘repair clause’ for component parts of complex products, however this clause is limited and remains to be seen how it will be interpreted in practice. A detailed examination of this new EU framework is outside the scope of this paper.

66 ibid, arts 4–6.

67 For an example of how design rights can be used in combination with patent rights for medical devices, see *Cantel Medical (UK) Ltd v ARC Medical Design Ltd* [2018] EWHC 345 (Pat), para 254; see also European Commission, Fact Sheet Intellectual property: Considerations For Medical Devices (October 2014) <https://www.fdanews.com/ext/resources/files/10-14/10-14-EU-IPR.pdf?1518220907> accessed 21 December 2023

68 In the EU, the applicable law is Directive 2015/2436 of the European Parliament and of the Council of 16 December 2015 [2015] OJ L 336/1. See also: Regulation (EU) 2017/1001 of the European Parliament and of the Council of 14 June 2017 on the European Union trade mark (EUTMR).

69 VK Ahuja, ‘Non-Traditional Trade Marks: New Dimension of Trade Marks Law’ (2010) European Intellectual Property Review 575; Irene Calboli, ‘Non-traditional Trademarks as Barriers to Competition, Innovation, and Creativity: What If Their Protection could be Effectively Limited in Practice?’ in Gustavo Ghidini and Valeria Falce (eds) *Reforming Intellectual Property* (Edward Elgar 2022) 1–17. It is conceded that registering non-traditional trademarks poses challenges, for example see recent decision in the context of colours as a trade-mark: Case T-187/19 Glaxo Group Ltd v EUIPO, General Court, 9th September 2020.

70 Martin Senftleben, ‘Signs Eligible for Trademark Protection in the European Union Dysfunctional Incentives and a Functionality Dilemma’ in Irene Calboli and Jane C Ginsburg (eds), *The Cambridge Handbook of International Comparative Trademark Law* (CUP 2020) 210.

71 For shapes there are limitations on the extent to which trademarks can apply, see art 7(1)(e)(ii) EUTMR. See also Case C-205/13 Hauck GmbH & Co KG v Stokke A/S C-205/13) Court of Justice of the European Union, 18 September 2014; Case C-48/09 Lego Juris v Office for Harmonization in the Internal Market (OHIM); Case C-299/99 Philips (EU:C:2002:377). See <https://guidelines.euipo.europa.eu/1803468/1788800/trade-mark-guidelines/3-shape-or-other-characteristics-of-goods-necessary-to-obtain-a-technical-result> accessed 14 May 2024.

72 Irene Calboli, ‘Beyond Patents: The Problems of Non-Traditional Trademark Protection for Medicines and Health Technologies’ (2020) 51 International Review of Intellectual Property and Competition Law 1, 2.

73 ibid.

74 ibid 4.

75 EU Trademarks are registered for 10 years, but can be renewed by further periods of 10 years each time, see <https://eur-lex.europa.eu/EN/legal-content/summary/european-union-trade-mark.html>

76 The trade secret and patent interplay is discussed in Tanya Aplin and John Liddicoat, ‘Discussion Paper on the Interplay between Patents and Trade Secrets in Medical Technologies’ (WIPO, October 2023) <https://www.wipo.int/edocs/mdocs/scp/en/wipo_ip_covid_ge_2_22/wipo_ip_covid_ge_2_22_paper.pdf> accessed 20 December 2023.

77 See Aplin and Liddicoat, ibid. For a discussion in the COVID-19 context, see Olga Gurgula and Luke McDonagh, ‘Access Denied: The Role of Trade Secrets in Preventing Global Equitable Access to COVID-19 Tools’ (19 June 2023). STOPAIDS & JUST TREATMENT (March 2023) <https://ssrn.com/abstract=4484507> accessed 20 December 2023; Olga Gurgula and Luke McDonagh, ‘On Compulsory Licensing of Trade Secrets to Safeguard Public Health’ (March 25, 2024). Available at SSRN: https://ssrn.com/abstract=4771745 or http://dx.doi.org/10.2139/ssrn.4771745, accessed 4 February 2025.

78 For a discussion of continuous glucose monitor systems, see Joseph Roberts, Victoria Moore and Muireann Quigley, ‘Prescribing unapproved medical devices? The Case of DIY Artificial Pancreas Systems’ (2021) 21 Medical Law International 42.

79 McMahon (n 2); Aisling McMahon, “Accounting for Ethical Considerations in the Licensing of Patented Biotechnologies and Health-Related Technologies: A Justification” in Naomi Hawkins (ed), *Patenting Biotechnological Innovation: Eligibility, Ethics and Public Interest* (Edward Elgar 2022), 163–195.

80 Arvind and McMahon (n 43).

81 ibid.

82 McMahon (n 16).

83 For a discussion in the COVID-19 vaccine context, see Siva Thambisetty and others, ‘The TRIPS Intellectual Property Waiver Proposal: Creating the Right Incentives in Patent Law and Politics to end the COVID-19 Pandemic’ (24 May 2021), LSE Legal Studies Working Paper No. 06/2021.

84 EU competition law, particularly art 102 TFEU may be relevant in certain ‘refusal to license’ contexts. However, competition law remedies are only available in exceptional circumstances, see Giorgio Monti, *EC Competition Law* (CUP 2007) 230. In the context of strategic accumulation of patents, see Olga Gurgula, ‘Strategic Accumulation of Patents in the Pharmaceutical Industry and Patent Thickets in Complex Technologies—Two Different Concepts Sharing Similar Features’ (2017) 48 International Review of Intellectual Property and Competition Law 385.

85 For a discussion of costs of exoskeletons in the USA, see Katleho Limakatso, ‘Exoskeletons: Costs and Where to Buy One Available’ (Healthnews, 17 May 2023) <https://healthnews.com/family-health/rehabilitation/exoskeletons-costs-and-where-to-buy-one/#:∼:text=Key%20takeaways%3A,price%20between%20%2470%2C000%20and%20%2485%2C500> accessed 11 December 2023

86 Ashraf S Gorgey, ‘Robotic Exoskeletons: The Current Pros and Cons’ (2018) 9 World Journal Orthopedics 112.

87 Jason Schwartz, ‘A Brand-New Kick’ (Bostonmagazine.com, 2013) <https://www.bostonmagazine.com/health/2013/11/26/prosthetics-research-boston-biom-ankle-prosthetic> accessed 1 December 2023.

88 ibid.

89 Joel Hruska, ‘A New Budget Exoskeleton Could Help Paraplegics Walk at a Drastically Lower Price’ (extremetech.com, 2016) <https://www.extremetech.com/extreme/222396-a-new-budget-exoskeleton-could-help-paraplegics-walk-at-a-drastically-lower-price> accessed 4 February 2025.

90 Arjun Kharpal, ‘The Wearable Robot that Helps People Walk Again’ (cnbc.com. 2015) <https://www.cnbc.com/2015/04/29/the-bionic-suit-that-helps-paralyzed-people-walk-again.html> accessed 1 December 2023.

91 National Institute for Health and Care Excellence, ‘EKSO Exoskeleton for Rehabilitation in People with Neurological Weakness or Paralysis’ (2017) <https://www.nice.org.uk/advice/mib93/resources/ekso-exoskeleton-for-rehabilitation-in-people-with-neurological-weakness-or-paralysis-63499466712517#:∼:text=The%20cost%20of%20the%20Ekso,ongoing%20maintenance%20and%20support%20costs> accessed 1 December 2023.

92 Signe Brewster, ‘This $40,000 Robotic Exoskeleton Lets the Paralyzed Walk” (MIT Technology Review, 1 February 2016) <https://www.technologyreview.com/2016/02/01/163493/this-40000-robotic-exoskeleton-lets-the-paralyzed-walk/accessed> 11 December 2023. There are lower priced exoskeletons available for commercial use for workers; however, such devices tend to be passive exoskeletons and not for health or rehabilitative uses. See Saheli D Burton, ‘Responsible Use of Exoskeletons and Exosuits: Ensuring Domestic Security in a European Context’ (2020) 11 Padalyn, Journal of Behavioral Robotics 370.

93 For a more recent discussion, see Emanuele Palazzi and others, ‘An Affordable Upper-Limb Exoskeleton Concept for Rehabilitation Applications’ (2022) 10 Technologies 22; Leslie Mertz, ‘The Next Generation of Exoskeletons: Lighter, Cheaper Devices are in the Works’ (2012) 3 IEEE Pulse 56.

94 See Carlos M Correa, ‘Guide for the Granting of Compulsory Licenses and Government Use of Pharmaceutical Patents’ (2020) 107 The South Centre Research Paper 13

95 McMahon (n 16); Aisling McMahon (2020) ‘Patents, Access to Health and COVID-19: The Role of Compulsory and Government-use Licensing in Ireland’ 71 Northern Ireland Legal Quarterly 331.

96 Paris Convention for the Protection of Industrial Property 1883, as amended.

97 [2018] EWHC 1256 (Pat); *GlaxoSmithKline UK Ltd v Wyeth Holdings LLC* [2017] EWHC 91 (Pat); The role of ‘public interest’ considerations in the patent injunction context for medical devices, is examined in: Philip Johnson, “The public interest and patent injunctions: Evalve v Edwards Lifescience [2020] EWHC 513 (Pat)” (2020) 10(3) Queen Mary Journal of Intellectual Property 392–400.

98 The court found: ‘The exception is justified by the need to protect the health of those patients for whom the Sapien 3 is the only suitable THV.’ [2018] EWHC 1256 (Pat), para 69.

99 [2018] EWHC 1256 (Pat), para 67.

100 ibid, para 42.

101 ibid, paras 42–58.

102 ibid, para 56.

103 See discussion also in Quigley and Aydihongbe, n 3, p. 301.

104 In the UK, s 60, Patents Act 1977, as amended is of relevance in terms of what constitutes infringement. For a discussion of how ‘making the product’ is construed for such repair purposes in the UK, see *Schutz (UK) Limited v Werit (UK) Limited* [2013] UKSC 16, if the part in question is viewed as an essential part of the invention, remaking that part for an existing device, would likely be an infringing act.

105 See also discussion in Quigley and Ayihongbe (n 3) 300; Leanne Wiseman and Kanchana Kariyawasam, ‘Restoring Human Dignity: Some Reflections on the Right to Repair & Medical Devices and Assistive Technologies’ (2022) 10(2) Griffith Journal of Law and Human Dignity 1–17; Kayleen Manwaring, ‘Slowing Down the Loop: Smart Devices and the Right to Repair’ (2024) 38 International Review of Law, Computers & Technology 268.

106 Eliza Strickland and Mark Harris, ‘Their Bionic Eyes are Now Obsolete and Unsupported’ (IEEE Spectrum, 15 April 2022) <https://spectrum.ieee.org/bionic-eye-obsolete> accessed 1 December 2023.

107 ibid.

108 ibid; Jane Wakefield, ‘Bionic Eyes: Obsolete Tech Leaves Patients in the Dark’ (*BBC News*, 17 February 2022) <https://www.bbc.com/news/technology-60416058> accessed 11 December 2023; Andrea Park, ‘Fantastic Technology and a Lousy Company’: Second Sight’s ‘Bionic Eyes’ Have Gone Obsolete While Still Implanted, Report Finds’ (*Fierce Pharma*, 17 February 2022) <https://www.fiercebiotech.com/medtech/second-sights-bionic-eyes-have-gone-obsolete-while-still-implanted-report-finds> accessed 11 December 2023; Eliza Strickland and Mark Harris, ‘Should Right-to-Repair Laws Extend to Bionic Body Parts? An IEEE Spectrum Exposé on a Bionic Vision Company Raised Troubling Questions’ (IEE Spectrum, 6 April 2022) <https://spectrum.ieee.org/bionic-right-to-repair> accessed 11 December 2023; Anders Sandberg, ‘Your Eyes will be Discontinued: What are the Long-term Responsibilities for Implants?’ (*Oxford Practical Ethics Blog*, 18 February 2022) <http://blog.practicalethics.ox.ac.uk/2022/02/your-eyes-will-be-discontinued-what-are-the-long-term-responsibilities-for-implants/accessed> accessed 11 November 2023.

109 ibid.

110 McMahon (n 2).

111 ibid.

112 See broader discussions in Andrea J MacNeill and others, ‘Transforming the Medical Device Industry: Road Map to a Circular Economy’ (2020) 39 Health Affairs 2088; Rob Lawlor, ‘Delaying Obsolescence’ (2015) 21 Science and Engineering Ethics 401.

113 See discussion in Kevin O’Reilly, ‘Manufacturers Brick Medical Devices, Too (PIRG)’ (15 May 2023) <https://pirg.org/articles/manufacturers-brick-medical-devices-too/> accessed 21 December 2023.

114 In some cases, there are health and safety reasons for not offering upgrades for existing devices, and liability issues that manufacturers may be concerned about if they facilitate third-party repairs. These are beyond the scope of this article.

115 See discussion in Strickland and Harris (n 107).

116 This builds on the discussion in Quigley and Aydihongbe (n 3) of how tangible and intangible rights and other legal considerations can impact device users, including impacting how device users modify their devices. Here this section focuses specifically on the impacts of IPRs only in this context. For a broader discussion of how IPRs can impact a range of technologies related to how we treat, use and modify the body, and the bioethical implications that can arise see McMahon (n 2).

117 Sophie Templer, ‘Closed-Loop Insulin Delivery Systems: Past, Present, and Future Directions’ (2022) 13 Front Endocrinol (Lausanne) 919942.

118 Roberts and others (n 78); Rachael Dickson and others, ‘#WeAreNotWaiting DIY Artificial Pancreas Systems and Challenges for the Law’ (2022) 39 Diabetic Medicine 1.

119 <https://openaps.org/what-is-openaps> accessed 21 December 2023.

120 Quigley and Ayihongbe (n 3); Jessica Bell and others, “#WeAreNotWaiting DIY Artificial Pancreas Systems and Challenges for the Law” (2022) 39 Diabetic Medicine 1; Karen Dimentstein, Jay M Sosenko and Kenneth W Goodman, ‘Do-It-Yourself Diabetes Management: Perspectives of a Patient, a Physician, and an Ethicist’ (2022) 40 Clinical Diabetes 70; L Heinemann and K Lange, ‘“Do It Yourself” (DIY)-Automated Insulin Delivery (AID) Systems: Current Status From a German Point of View’ (2020) 14 Journal of Diabetes Science and Technology 1028.

121 For UK, see s 60(5) of the Patents Act, 1977, as amended.

122 On the intersection between IPR and the Right to Repair: Anthony D Rosborough, Leanne Wiseman and Taina Pihlajarinne, ‘Achieving a (Copy)right to Repair for the EU’s Green Economy’ (2023) 18 Journal of Intellectual Property Law & Practice 344.

123 Sean M Ragan, ‘I’m Just a Girl Living with Type 1 Diabetes Who Got Tired of Waiting’ (proto.life, 2018) <https://proto.life/2018/08/medicine-ignored-this-insulin-problem-hackers-solved-it> accessed 23 August 2023.

124 ibid.

125 ibid. See also Sarah Zhang, ‘People are Clamoring to Buy Old Insulin Pumps’ (theatlantic.com, 2019) <https://www.theatlantic.com/science/archive/2019/04/looping-created-insulin-pump-underground-market/588091/> accessed 1 December 2023.

126 This is discussed in Aisling McMahon and Opeyemi Kolawole, ‘Intellectual Property Rights and Control over the Repair of Medical Devices: The Need (and Challenges) around Rights to Repair’ (Working Paper 2024, on file with authors).

127 Dickson and others (n 117). Quigley and Aydihongbe (n 3) who discuss legal challenges posed by biohacking in the ‘everyday cyborg’ context, 296–298.

128 These are examined in detail in McMahon and Kolawole (n 125).

129 On device-user’s needs, see Joseph TF Roberts, Victoria Moore and Muireann Quigley, ‘Prescribing Unapproved Medical Devices? The Case of DIY Artificial Pancreas Systems’ (2021) 21 Medical Law International 42; see also Benjamin J Louviere, ‘Tune UP in America’s Healthcare Market: Securing the Right to Repair for Medical Devices’ (2023) 48 Journal of Corporation Law 183.

130 There are limited patent exemptions allowing patented technologies for research purposes, but generally, such exemptions do not apply if the research is to develop a technology for commercial purposes. The research exemption is discussed around patents and genome editing technologies: Duncan Matthews and others, ‘The Role of Patents and Licensing in the Governance of Human Genome Editing: A White Paper’ (30 July 2021), Queen Mary Law Research Paper No. 364/2021 <https://ssrn.com/abstract=3896308> accessed 23 June 2024.

131 For a discussion see Gurgula (n 84); Michael A Heller, ‘The Tragedy of the Anticommons: Property in the Transition from Marx to Markets’ (1998) 111 Harvard Law Review 621; Carl Shapiro, ‘Navigating the Patent Thicket: Cross Licenses, Patent Pools, and Standard-Setting’ in Adam B Jaffe and others (eds), *Innovation Policy and the Economy* (National Bureau of Economic Research 2001) 120.

132 Reed F Beall and others, ‘Patent “Evergreening” of Medicine-Device Combination Products: A Global Perspective’ (2022) 18 Healthcare Policy 14.

133 See discussion in the COVID-19 context, in Thambisetty and others (n 12).

134 We discuss this in detail elsewhere, see McMahon and Kolawole (n 125).

